# Livelihood vulnerability household fishermen household due to climate change in Lampung Province, Indonesia

**DOI:** 10.1371/journal.pone.0315051

**Published:** 2024-12-31

**Authors:** Maya Riantini, Maesti Mardiharini, Saptana, Bedy Sudjarmoko, Eka Kasymir, Lestari Gita Nur’aini, Salsa Hentia Anindita, Mat Syukur, Armen Zulham, Budi Wardono, I. Ketut Ardana, Chandra Indrawanto, Agus Wahyudi

**Affiliations:** 1 Study Program of Agribusiness, Faculty of Agriculture, University of Lampung, Bandar Lampung, Indonesia; 2 National Research and Innovation Agency, Jakarta, Indonesia; 3 Research Center for Cooperative, Corporation and People’s Economy, National Research and Innovation Agency, Jakarta, Indonesia; 4 Undergraduate Program of Agribusiness, Faculty of Agriculture, University of Lampung, Bandar Lampung, Indonesia; CIFRI: Central Inland Fisheries Research Institute, INDIA

## Abstract

The livelihood of small-scale fishers is highly dependent on marine resources and coastal areas while the condition of marine waters is increasingly unpredictable due to seasonal uncertainty and extreme weather due to climate variability. This condition has a negative impact on fish catches and the income of small-scale fishermen. The study results were obtained through interviews with respondents using a structured questionnaire. Sampling was carried out using multistage random sampling based on the type and number of ships controlled (1 GT-5GT). The total sample of respondents interviewed was 166 fishing households, consisting of 36 respondents from Bandar Lampung City, 65 respondents from South Lampung Regency, and 65 respondents from Tanggamus Regency. Data was evaluated using three analysis methods, namely household income structure, indicators of income vulnerability, and adaptation mechanisms. Income structure and income vulnerability use a quantitative approach, while adaptation mechanisms use a qualitative approach. The results of this empirical study found that the source of income of traditional capture fisher households is from: fishing business (on the farm) averaged 82.22%, in Tanggamus Regency, the proportion reached 86.22%. The income vulnerability index of traditional capture fisher households (LVI-IPCC value) in Bandar Lampung City and South Lampung Regency is positive (0.39 and 0.36). The income vulnerability index of traditional fishermen in Tanggamus Regency is negative -0.29. Fishermen employ an adaptation mechanism that engaged the five fundamental facets of income capital, namely natural capital, human capital, physical capital, financial capital, and social capital.

## Introduction

Indonesia is one of the largest archipelagic countries in the world, with 17,508 islands that stand on 5.8 million km^2^ of marine waters [1]. This condition gives Indonesia the potential for abundant fisheries and marine resources. The marine and fisheries sector is also one of the economic sectors that play an important role in national economic development, especially in providing animal protein food, earning foreign exchange, providing employment, and providing a source of income for fishermen and fish farmers.

Lampung Province stands as a region endowed with substantial fisheries and marine resources, spanning the expanse of the East Coast of Lampung Province. Nevertheless, the past five years have witnessed a marked decline in the average production of capture fisheries within Lampung Province. This decline in production can be primarily attributed to the increasingly unpredictable state of sea waters within the Lampung region, which is rooted in the vagaries of seasonal variations and climatic patterns affecting Lampung waters.

The velocity of prevailing winds and the extent of precipitation notably shaped these fluctuations in seasonality and weather patterns. Referring to Lampung Province BPS data [2] it is known that the average wind speed in Lampung Province over the past five years has fluctuated. Wind speed caused by temperature and pressure in the atmosphere is one of the natural factors that affect the condition of Lampung waters. Wind direction and ocean currents from the South China Sea (South China Sea monsoon) and Indian Ocean Dipole affect the condition of waters in Sumatra such as in Lampung Bay [3] Wind direction, Mount Krakatau, monsoon winds, air temperature (land and ocean) affect rainfall. The rainfall of Lampung Province from November is also in the Medium-High criteria (151–400 mm/month) with normal (N) to above normal (AN) rainfall properties [4]. High rainfall can affect fishermen’s activities in fishing so that it will affect income. In addition to rainfall, wind direction also affects the condition of marine waters and the occurrence of extreme weather. This extreme weather condition is climate change. The uncertain condition of sea waters due to the influence of extreme weather affects the activities of fishermen in catching fish at sea and has an impact on reducing the catch of fishermen. This is in line with research [5], where the number of rainy days and the amount of rainfall affect the production of fish catches.

Global warming over the past century has resulted in climate change and extreme weather, most of which are indicated as environmental disasters such as the phenomenon of floods, droughts and shifts in the rainy season [6]. Rising temperatures, sea level rise, extreme rainfall and extreme winds are causing erosion of coastal areas and mangroves and degradation of marine resources. In turn, these issues impact small-scale fishers who rely heavily on weather stability to conduct their social and economic activities [7]. Extreme weather is an ecological symptom that arises as a result of climate change that affects the activities of fishermen at sea. Annual climate variability in Indonesia is generally described by a seasonal cycle, known as the Asia—Australia Monsoon Circulation System [8]. Climate variability can cause several impacts, such as unpredictable and erratic rainfall, seasonal shifts and uncertainty, changes in sea surface temperature, changes in wind patterns and high waves that can cause fishermen to fail to go to sea, as well as changes in rainfall patterns, flooding, and drought phenomena [9]. In general, fishers who depend highly on natural stability are heavily impacted by these changes; therefore, strengthening their adaptability is seen as the best response [10].

Seasonal changes that occur within a certain period of time have an impact that begins to be felt and becomes a threat to human life, especially fishermen whose lives depend on nature [11]. Climate change is also likely to have severe biological and economic consequences [12], climate change and pollution have a negative impact on fishing activities by fishermen. Fishermen report difficulties in meeting basic livelihood needs, including feeding families, paying children’s school fees, and paying hospital and utility costs [13]. Climate change and extreme weather have had the effect of reducing the level and stability of fishermen’s incomes [14]. The impacts of climate change on fisher households include difficulty predicting fishing seasons, changes in fishing locations, reduced fishing frequency and unpredictable fishing times, and decreased fish catches [15]. In addition, climate change also impacts the biology of fish and plankton life in terms of developmental rates, reproduction, behaviour and survival [16].

The results of the study by [17] revealed that over the past ten years, because of climate variability, about 20% of the heads of fishing households on the East Coast of Bangladesh have switched professions from fishing to non-capture fisheries, such as becoming pedicab drivers, small businesses, and food crop farming, which is increasing in the fishing villages studied. Based on 2011 and 2021 data in rural coastal areas in Indonesia, there is also a shift in business activities, namely from capture fishermen and pond farmers to rice farming and non-agricultural activities, which encourages the phenomenon of urbanization [18]. However, some fishermen are applying their traditional knowledge to cope with climate change pressures and preserve coastal biodiversity. Studies related to social vulnerability and community strategies to adapt to climate change in coastal Brazil found that remoteness and a lack of institutional support related to climate change increased the vulnerability of fishing communities [19].

The results of a study [20] in Zhoushan City, Zhejiang Province, China, showed that highly vulnerable fishers amounted to about 37.35%, and they had some unique characteristics such as advanced age and low education level. The livelihood vulnerability of fishermen engaged in recreational fishing is relatively low, while those engaged in non-fishing differ greatly from each other. It means that there has been a shift from capture fisheries and aquaculture activities to non-agricultural activities, with quite diverse activities.

The findings of the study, as determined through the utilization of the Tobit model as expounded by [21], reveal a robust influence of asset wealth, household composition, local institutional factors, and location-specific attributes inherent to the two studied villages on households’ choices to engage in diverse fisheries activities. In addition, the results revealed that ownership of fishing assets and the influence of access were the main determinants of variations in total household income. The results of the regression analysis showed that the number of household income sources, the impact of disturbance, and equipped fishery facilities can affect the livelihood vulnerability of fishers. It was found that small-scale fishers in both the upper and lower strata in Tegal Regency, Central Java Province, are less resilient to climate variability [22]. Upper-layer fishers use physical and financial capital for exploitation and spatial strategies. On the other hand, lower-layer fishermen use more of their social capital, such as group affiliation, building mutual trust, and a patron-client system based on the moral economy. The conceptual model of coastal village development in Donggala Regency, Central Sulawesi Province, that implements an ethical coastal area management approach integrated into the fisheries agribusiness system can have implications for community protection, income generation, and sustainable fisheries management [23].

Most fishing communities in Bandar Lampung, South Lampung Regency, and Tanggamus Regency use the proceeds from fishing as their main source of income. Climate variability affects water conditions that directly impact communities that depend on marine ecosystems. Supported again by the results of research [24], climate change has a tremendous impact on reducing the socio-economic status of fishermen, reducing their sources of livelihood, low-income levels, and poor health. Weather conditions over the past year in sea waters and coastal areas of Lampung can be said to be erratic, so there is uncertainty in fishing, and fishermen’s income has decreased. Traditional capture fishermen in the three districts are classified as small-scale fishermen and still use traditional fishing gear, making it difficult for fishermen to deal with the impacts of increasingly extreme climate variability. Therefore, a decrease in income because of the impact of climate variability has the potential to cause traditional capture fisher households in the three districts to experience vulnerability.

The problems of small-scale fisheries include low economic performance and limited ability or expertise to deal with global pressures, including climate change [22]. Livelihood vulnerability is a situation where individuals or households face pressures and shocks to their sources of livelihood or income that threaten their success and sustainability. The Intergovernmental Panel on Climate Change (IPCC) (2007) characterizes vulnerability as a function of three capacity components: adaptive capacity, sensitivity, and level of exposure. The current climate variability also affects the decline in fish catches, potentially causing vulnerability to the livelihood or income of fishing households living around the three districts.

The livelihoods of people in coastal areas are dynamic and highly dependent on nature, so they are vulnerable to climate change [25], dan [26], therefore, it is necessary to know how income vulnerability to climate change and how to adapt to it. Based on the description of the problems above, this study aims to analyze the income structure of fisher households, analyze income vulnerability, and analyze the adaptation mechanism carried out by fisher households in an effort to deal with the impact of climate variability. The results of this study are expected to provide an academic contribution on how fishermen adapt the strategies to extreme weather conditions due to climate variability to maintain the level and stability of fishermen’s income.

Traditional fishermen typically face significant limitations in capacity, financial resources, and flexibility, making fishing activities a source of socioeconomic risks characterized by potential income loss and uncertain outcomes. Nonetheless, these risks remain inadequately understood, especially from the vantage point of fisher. In this study, we addressed the risks and institutional challenges through three primary objectives. Firstly, we aim to comprehensively assess potential vulnerability associated with fishing activities on traditional fishing. This involves mapping out these risks and developing effective adaptation strategies with the specific focus on the role of fishermen’s perception and their influence on successful sustainability of income source. Secondly, we seek to identify and analyze critical parameters within the adaptation mechanism, utilizing these parameters to scrutinize elements that might contribute to fisher failures during climate change adaptation. Finally, we delve into the key factors influencing the performance and effectiveness of government facilitation, including funding mechanisms, infrastructure, and institutional policies. Through these research objectives, we aspire to provide valuable insights and evidence-based recommendations with the potential to impact the Indonesian climate change adaptation. This impact will be particularly evident in the enhancement of fishing productivity and the effectiveness of institutional policy in supporting traditional fishing.

## Materials and methods

The object of this research is households whose main source of income is traditional capture fishermen. The object of this research is households whose livelihood is fishing in areas affected by the climate change in Lampung Province. Indicators of climate change refer to [27] Economic losses due to extreme climate events can be evaluated from their impact on main job, and prices of some commodities. Disasters reduce work productivity, especially if the main jobs of society are vulnerable to disasters [28].

**Fig 1:**
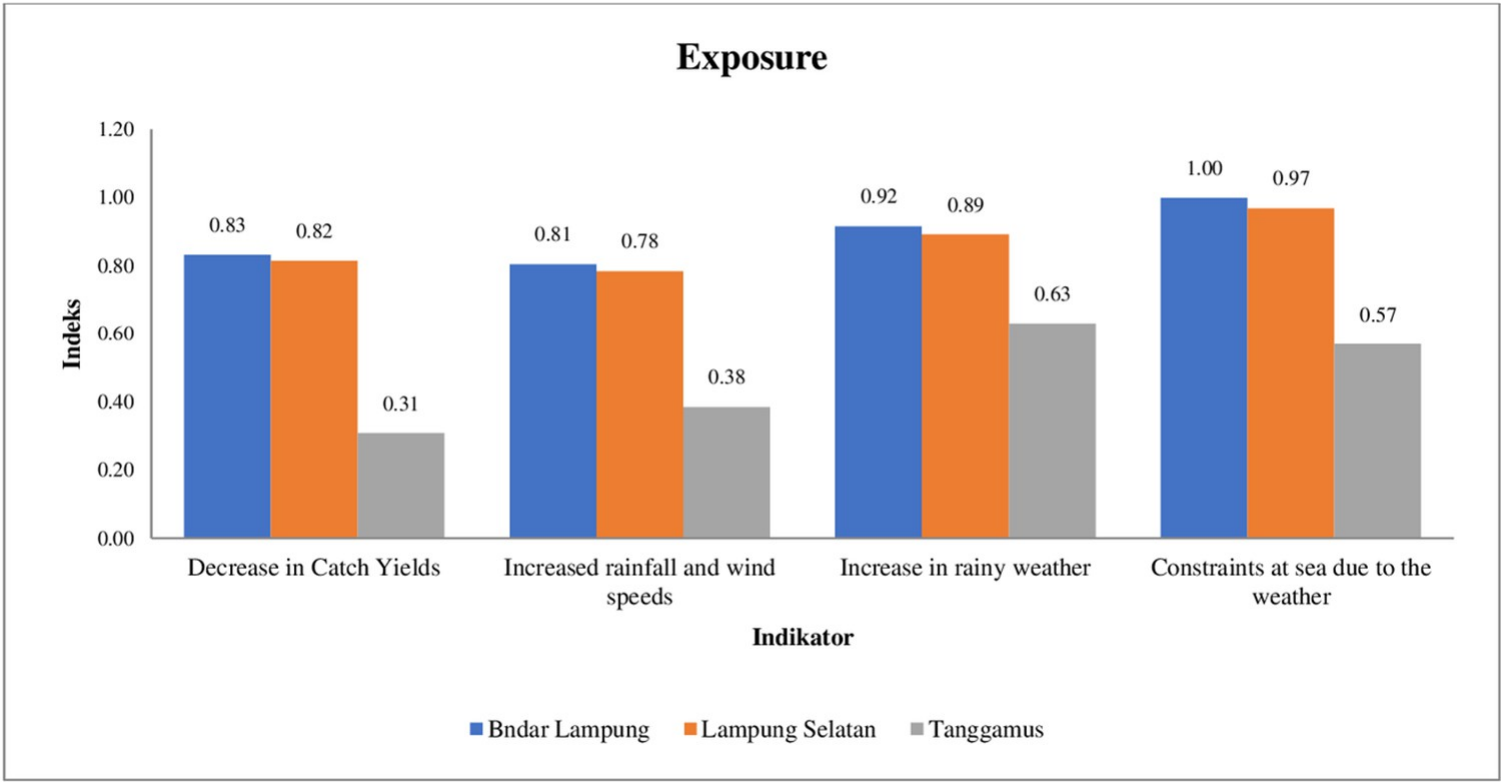
Fishing households’ dispersion index.

The object of this research is households whose main source of income is traditional capture fishermen. The population in this study is coastal village fisher households in Bandar Lampung City, South Lampung Regency, and Tanggamus Regency, the complete research location can be seen in Fig 1. This study used a multistage sampling procedure, adopting research by [29]. First, the location was selected purposively with the consideration that the majority of the population is fishermen and has a Fish Auction Place (TPI) so that Bandar Lampung City, South Lampung Regency and Tanggamus Regency, Lampung Province, Indonesia were selected.

The selection of fishermen based on their households, using stratified sampling based on the number and type of boat ownership. Furthermore, the respondents to this study were chosen using simple random selection. Determination of the number of respondents using the Slovin formula, as follows [30] with a total of 166 fishermen from traditional fishing households that possess an average small boat ranging in size from 1 GT to 5 GT. The sampling information are as follows 36 fishing households from Bandar Lampung, 65 fishing households from South Lampung Regency and 65 fishing households from Tanggamus Regency.

Primary data is obtained through direct interviews with respondents who meet the research criteria using questionnaires and direct observation and recording related to the data used in the study. Secondary data is data based on literature relevant to research and data from relevant agencies. Validity and reliability tests were conducted on the vulnerability section of the questionnaire. The validity value can be determined by finding the r count and comparing it with the r table. Question items are said to be valid if r count > r table. The reliability value can be good if Cronbach’s Alpha value is greater than 0.60 [31].

The data analysis method to analyze the income structure of fisher households is based on the household income structure. The income structure analysis in this study refers to the calculation of total income of fisher households. The source of income of fisher households can be categorized as on fisheries income, Income from fishing hired labourers and non-fisheries income, such as crop farming and non-agricultural activities (industry, trade and services).

To analyse capture fisheries business income is calculated using the formula according to [32]:

π=TR–TC (1)

where,

TR=P.QandTC=TFC+TVC

Description:

π = Income (IDR/season)

TR = Total revenue (IDR/season)

TC = Total cost (IDR/season)

P = Product price (IDR/kg)

Q = Total production (kg)

TFC = Total fixed costs (IDR/season)

TVC = Total variable cost (IDR/season)

IDR = Indonesia Rupiah

Furthermore, to calculate the household income of traditional capture fishermen in Bandar Lampung City, South Lampung Regency, and Tanggamus Regency, the following formula was used.

Prt=Ponfisheries+Pofffisheries+Pnonfisheries (2)

Description:

P_rt_ = Fishermen’s household income (IDR/year)

P_*on fisheries*_ = Capture fisheries business income (IDR/year)

P_*off fisheries*_ *=* Income from fishing hired labourers (IDR/year)

P_*non fisheries*_ *=* Business income excluded from the fisheries sector (IDR/year)

The income vulnerability analysis of fisher households in this study uses the Livelihood Vulnerability Index (LVI). According to several researchers [33–36], the LVI is calculated by applying a balanced average approach in which each main component comprises several sub-components. However, each different sub-component contributes equally to the overall index.

Analysis of the vulnerability index of fishing households uses the Livelihood Vulnerability Index (LVI). LVI measurements were carried out to see the value of fishermen’s household income vulnerability. Refer to the Intergovernmental Panel on Climate Changes, LVI measurements are divided into three categories including exposure, sensitivity, and adaptive capacity. Exposure consists of economic and ecological impact components. Adaptive capacity consists of components, namely natural capital, human capital, physical capital, financial capital and social capital. Sensitivity consists of two sub components, namely food and boat ownership. The main components, sub-components, and components measured in this study can be seen in Table 1.

**Table 1:** Main components, sub-components, and components measuring vulnerability of fisher households in Lampung Province.

Main Component	Sub-component	Measured Component
Exposure	Economic and ecological impacts	• Percentage of households (RT) experiencing < catches • Percentage increase in rainfall and wind speed in the last 5 years • Percentage of households experiencing an increase in severe weather phenomena in the last 1 year • Percentage of households experiencing problems in fishing activities
*Adaptive Capacity*	Natural capital	• Percentage of households with longer duration at sea
	Human capital	• Percentage of household heads who completed primary school • Percentage of household members who attend school • Percentage of RT heads whose main livelihood is fishing • Percentage of neighbourhood heads who have a second job • Percentage of households over 50 years old • Percentage of working families
	Physical capital	• Percentage of household that have facilities to support fishing operations (GPS/compass) • Percentage of households owning other fishing gear
	Financial capital	• Percentage of households with savings • Percentage of households receiving remittances from relatives/children • Percentage of households with other sources of income,
	Social capital	• Percentage of fishing households that lend money to other fishers • Percentage of fisher households receiving assistance from the government in the past year • Percentage of households borrowing from lenders/renters/cooperatives • Percentage of households whose selling price is determined by a third party
*Sensitivity*	Food	• Percentage of households whose food depends on catch
	Ship ownership	• Percentage of households that use part of their catch for the daily consumption

source: [33]

In this study the LVI was calculated by applying a weighted average approach (Hahn, et al. 2009) whereby each of the main indicators consists of a number of different sub-indicators, but each of the different sub-indicators contributes equally to the overall index. Therefore, the formula used to measure the livelihood vulnerability index is:

$$Index\;Sd=\frac{Sd-Smin}{Smax-Smi}$$ (3)

Description:

Sd = Sub-component value

Smin = Minimum value of the sub-component

Smax = Maximum value of the sub-component

Thenceforth, determine the value of the main component, namely by using the formula:

$$Md=\frac{\sum_{i=1}^{n}index\;Sdi}{n}$$ (4)

Description:

Md = Main component values

Indeks Sd = Sub-component value

n = The number of sub-components

Then, determine the index value of the LVI contribution factor by using the formula:

$$CFd=\frac{\sum_{i=1}^{n}Wmi.Mdi}{\sum_{i=1}^{n}Wmi}$$ (5)

Description:

CFd = LVI contribution factor index

Wmi = Sub-component weights of main indicators

Mdi = Main component values

Finally, the formula used in calculating the level of vulnerability of fishing households is the LVI calculation formula used based on the Intergovernmental Panel on Climate Change (IPCC), namely:

$$LVI(IPCC)=(e_{d}-a_{d})x\;s_{d}$$ (6)

Description:

LVI = Livelihood Vulnerability index

e_d_ = Fishermen exposure index

a_d_ = Adaptive capacity index of fishermen

s_d_ = Fishermen sensitivity index

Therefore, the researchers use the qualitative descriptive analysis method to analyze the adaptation mechanism. Researchers used qualitative descriptive analysis to understand how Indonesian fisher households in Bandar Lampung, South Lampung District, and Tanggamus District, Lampung, Indonesia, adapted to environmental changes. The researchers looked at different factors, like their resources and how they used them to survive. Data analysis related to the adaptation mechanism of fishing households to climate change in the form of extreme weather or seasonal changes uses qualitative data analysis procedures [37] to find adaptation mechanisms by fishing households by identifying certain patterns through a series of data and information that has been collected [38]. The application of qualitative analysis techniques is intended as an analysis from the researcher’s perspective to examine the extent to which the phenomenon of adaptation mechanisms of fishing households occurs by revealing qualitative data in detail to find the relationship between one pattern and another [39].

## Results

### Symptoms of climate change in Lampung

Symptoms of climate change occurring in Lampung are based on three indicators [27], namely: (1) changes in air temperature, (2) changes in rainfall; and (3) the history of events related to climate change. It was stated that at the last 23 years (1998–2020) there was a decrease in minimum temperature of 0.313oC/year and a decrease in maximum temperature of 5.152oC/year. It was further stated that there had been changes in rainfall outside the usual average rainfall in both the rainy season and the dry season, respectively with a decrease in rainfall in the rainy season of 0.804 mm/year and an increase in rainfall in the dry season of 0.419 mm/year. These two events had an influence on the sea level rise, which at certain times caused tidal flood disasters in areas directly adjacent to the coastline. Apart from that, changes in air temperature also affect wind speed. Referring to Lampung Province BPS data [2], it is known that the average wind speed in Lampung Province over the past five years has fluctuated. Wind speed is one of the natural factors that affect water conditions in the Indonesian region. The speed of the wind that blows indirectly determines the size of the sea waves. Wind speed also affects the pattern and distribution of rainfall. Rainfall in Lampung Province from November is also in the Medium-High criteria (151–400 mm/month) with normal (N) to above normal (AN) rainfall properties [2]

### The structure of fishermen’s household income in Lampung Province

The income structure of fisher households in Lampung Province is methodologically analyzed based on sources of income from capture fisheries and excluded capture fisheries. The income of fisher households is a comprehensive aggregation of the earnings generated by each constituent member of the family living within the fisher household over a specified temporal period. Adopting the results of research [36], the sources of income of fisher households are divided into three categories: income from fishing businesses (on fisheries), income in the agricultural sector excluded from capture fisheries (off fisheries), and income excluded from capture fisheries (non-fisheries). such as crop farming, industrial businesses, non-fishing service trade

The source of income of fisher households in one year (on fisheries), with an average percentage of fisheries income of 82.22% in the three districts. In contrast, the remaining fisheries income of 4.07% comes from activities as fishing laborers, and non-fisheries income of 13.71% is obtained from activities excluding the fisheries sector, such as construction workers, security guards, village health workers of Integrated Healthcare Center (Posyandu), employees, household assistants, tailors, the teacher of the Koran (Islamic teacher), and traders (stalls). The Tanggamus district has the largest percentage, followed by Bandar Lampung, and South Lampung. The fisheries income of fisher households comes from the sale of the entire fish catch. It shows that fishing is still the main job in meeting the needs of daily life. The results are under research [40], which concluded that income from fishing has the highest contribution to torch fishermen households in Bandar Lampung City. The results are also similar to those of [41]. The capture fisheries business is still the mainstay business sector undertaken by coastal fishing communities in Kota Agung, South Sumatra Province, in meeting household living needs. It differs slightly from the results of research [42], where the largest source of income for tourist fishermen households comes from the non-fisheries sector, namely the tourism business. Overview of the income structure of fishermen’s households in Lampung Province is shown in Table 2.

**Table 2:** Presentation of income structure (earnings) of fisher households in Lampung Province (%).

Types of Income	Bandar Lampung	South Lampung	Tanggamus	Average
Capture fisheries income (*on fisheries*)	81,19	79,26	86,22	82,22
Income from fishing hired labourers (*off fisheries*)	5,32	4,94	1,94	4,07
Income excluded the fisheries sector (*non fisheries*)	13,49	15,8	11,84	13,71
**Total**	**100**	**100**	**100**	**100**

Empirically in the field, aquaculture activities were only found in a small number of fisher household respondents, for example in the research location of Bandar Lampung City, fisher households were found to cultivate grouper fish and in Tanggamus District, seaweed cultivation was found. The prospect of aquaculture can be an alternative source of income for fisher households, especially those affected by climate change.

### Income vulnerability of fisher households in Lampung Province

Vulnerability is the degree or level of susceptibility or inability to cope with the adverse impacts of a disaster event [43]. In the context of climate variability, [44] define *vulnerability* as the state of a livelihood system that cannot cope with the various impacts of climate variability, such as the level of sensitivity and lack of adaptive capacity to adjust. According to the [45], the level of income vulnerability of fishing households is measured through three components, namely exposure, adaptive capacity, and sensitivity.

#### Exposure

Coastal social-ecological systems are vulnerable to climate change, with impacts spreading unevenly among human communities [46]. Adger (2006) in [47] says exposure is the magnitude and duration associated with climate problems. Fisher households are exposed to stress due to climate issues that affect their livelihoods. In fisheries-based livelihoods, exposure means the extent to which the livelihood system is exposed to the significant impacts of climate variability [33] in [34]. Fishing households’ dispersion index is shown in Fig 1.

**Fig 2:**
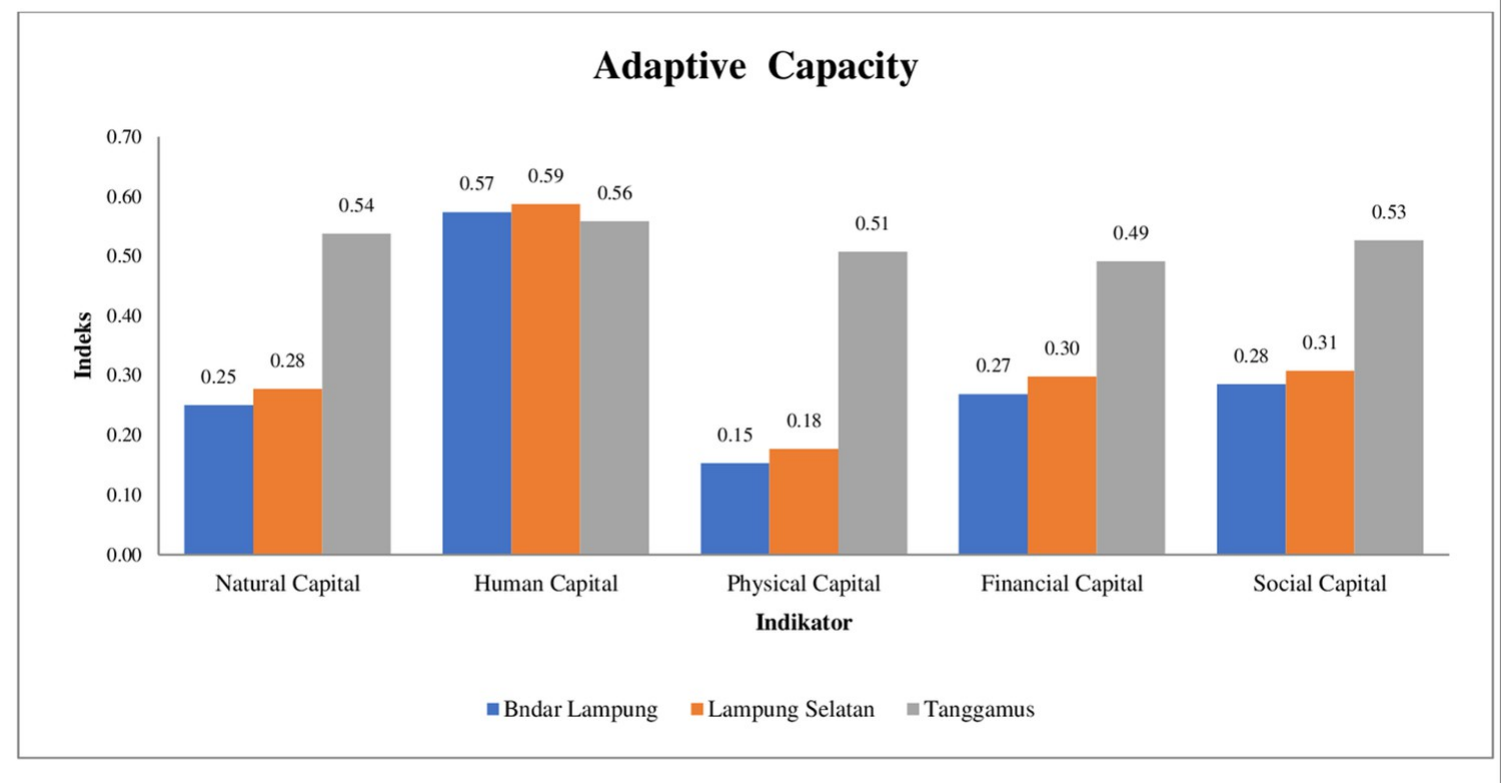
Adaptive capacity index of fishing households.

Fig 2 shows the calculation of the exposure index of traditional capture fisher households in Bandar Lampung City, South Lampung Regency, and Tanggamus Regency. Exposure is measured through the sub-components of decreased fish catch, increased rainfall and wind speed over the past five years, increased bad weather over the past year, and constraints on fishing due to bad weather. Almost all fisher households experienced a decrease in catches in the past year due to an increase in bad weather in sea waters due to increased rainfall, wind, speed, and high waves in Lampung Sea waters. It is supported by the results of research [48] that the amount of fish production caught by fishermen has decreased because of the impact of the tsunami in southern Lampung Regency.

This condition is an obstacle for fishermen in carrying out fishing business activities at sea. Fishermen in this study are classified as traditional fishermen who still use small boats (1 GT—5 GT) and very simple technology so that it is still difficult to adapt in the face of extreme weather. The calculation results show that the level of exposure in terms of ecology and economy of fishing households in three research locations is on average high at 0.74. With an average exposure index of several indicators, Bandar Lampung City is the highest at 0.89, followed by South Lampung Regency at 0.87, and finally Tanggamus Regency which has the lowest exposure index at 0.47. This shows that Tanggamus Regency is a regency that is relatively able to survive the exposure of ecological and economic aspects, this condition is supported by the results of the study [49] which states that fishermen in Tanggamus Regency are more modern, especially in the use of boats and fishing gear.

The high level of exposure indicates that climate variability causes shocks to the main source of income of traditional capture fisher households in Bandar Lampung, South Lampung, and Tanggamus. The results of this study are also in accordance with research [50] where the exposure index values of both groups of fishing households are quite high, namely Tangsi fishermen 0.53 and wave fishermen 0.64. Therefore, to reduce the impact of exposure on traditional fishermen, modernization of fishing gear, improvement of technical skills at sea, and managerial capabilities of capture fisheries businesses are needed

#### Adaptive capacity

Households respond to shocks or stresses from climate variability with various adaptation strategies. Responses to stress can be divided into three categories, namely, adapt, react, and coping strategies [51]. Adaptation capacity is the ability of a system or individual to adjust and use opportunities to overcome or reduce potential damage because of the pressure of climate variability [52]. Adaptation needs to be done to deal with changes that occur, one of which is climate change, such as thorough diversification of income sources, diversification of fishing gear, migration to cities, and development of social networks among fishermen [53]. One of the social and economic network developments that are considered quite strategic is through a network of business partnerships that are mutually needy, beneficial, and strengthening. Adaptive capacity index of fishing households is shown in Fig 2.

**Fig 3:**
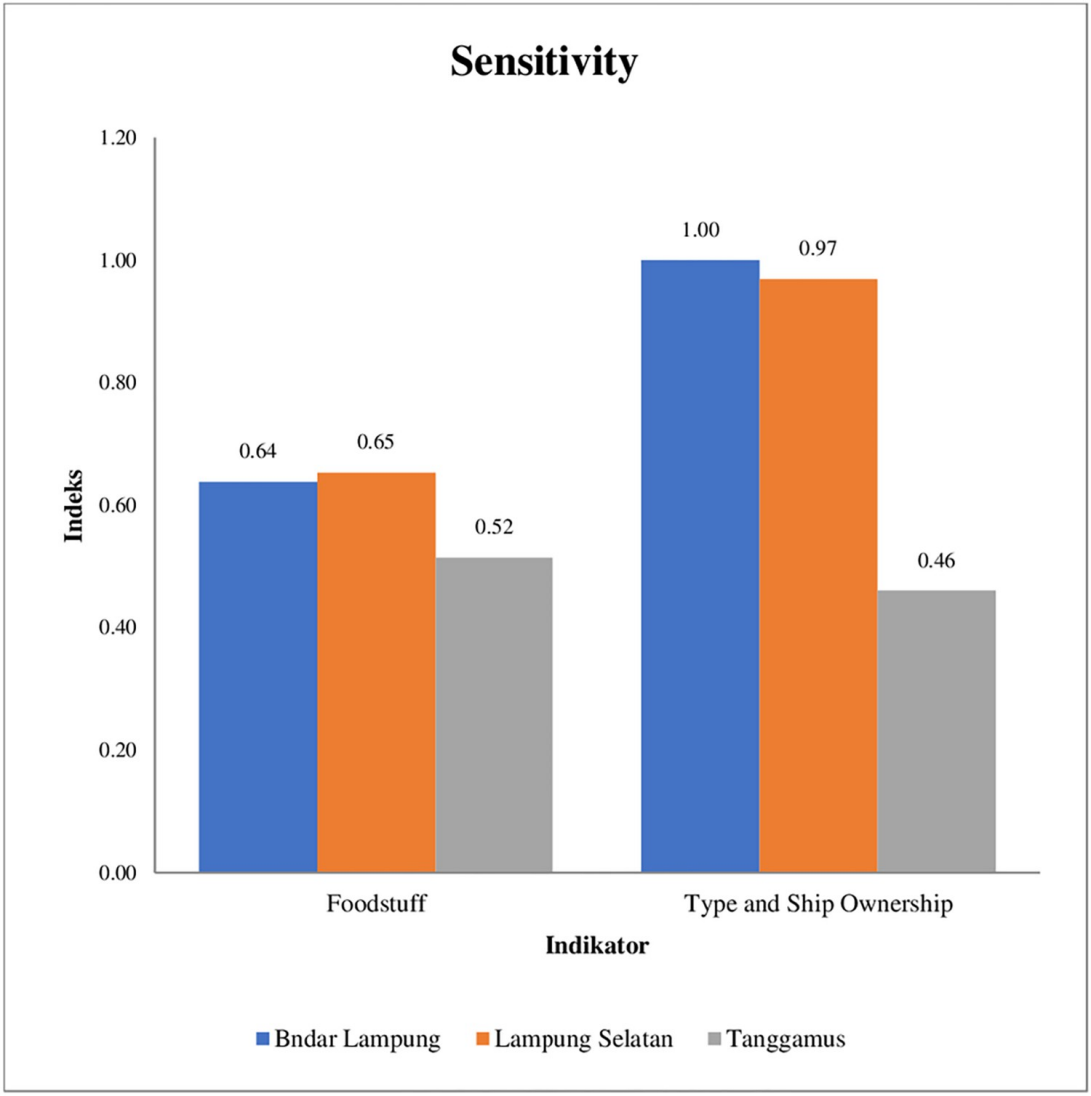
Sensitivity index of fishing households.

Fig 3 shows the results of the calculation of the adaptive capacity index of fisher households in the research location. The 5 indicators of fishermen’s adaptive capacity, the human capital indicator is an adaptation strategy of small-scale fishermen responding to extreme weather that occurs in Lampung waters. This adaptation strategy is carried out because the social system in the community is still strong, as well as the kinship system in the family. The form of the small-scale fishermen’ community is to provide mutual assistance when there are relatives who are in trouble. Meanwhile, the form of the family is to make maximum use of the labor potential of household members to support family finances. The existence of working family members helps to maintain survival in the face of declining income due to climate variability. The results of this study are different from those of [54], where ecotourism fishing households in Nagari Mandeh, in South Pesisir Regency, West Sumatra Province are more ideal in utilizing natural capital, social capital, and physical capital compared to the utilisation of financial capital and human capital which are classified as weak.

Small-scale fishers in Lampung are relatively non-adaptive to the other 4 indicators, due to various factors, such as; the low level of education and experience working as a fisherman so that they do not have the ability to improve skills to other livelihood sources, productive assets, and work networks with parties outside the village. These factors are suspected to be obstacles to small-scale fishers being unable to utilize the natural capital of marine and coastal services to support their household livelihoods. The age of respondents is estimated to be very influential on physical capital indicators, except in Bandar Lampung and South Lampung, the physical capital of small-scale fishers in Tenggamus adapts to extreme weather changes, family members of Tenggamus fishers are estimated to work temporarily outside the village in activities outside fishing.

The adaptive capacity of small-scale fishing households in the research location is low. This is because fisher households are still under limited availability and access to resources, so they have not been able to utilize the five income capitals optimally. However, Tanggamus Regency has the highest capacity index compared to other research locations. In relation to fishermen who have not been able to utilize the five capitals optimally. Small-scale fisher households in Tenggamus can do temporary work outside of fisheries activities, because they have a work or kinship network (social capital) outside Tenggamus. This is because they know each other, trust each other, thus forming social connections. This social connection makes small-scale fishing households in Tanggamus have better financial capital adaptation compared to small-scale fishermen in South Lampung and Bandar Lampung. Research results by [34]. A lack of adaptive capacity in terms of diverse physical, natural and financial capital shapes livelihood vulnerability. Furthermore, [55] stated that fishing groups with higher social adaptive capacity are considered less vulnerable as a whole.

#### Sensitivity

*According to* [56] states that sensitivity analysis can determine the behavior of the model in response to changes in a variable. *Sensitivity* is defined as the extent to which the system responds to impacts that occur because of climate variability, including adverse and beneficial impacts [57, 58] state that the lower the sensitivity of the system, the better it can withstand the effects of climate change without adaptation efforts. The sensitivity index of fishing households to climate variability is shown in Fig 3.

**Fig 4:**
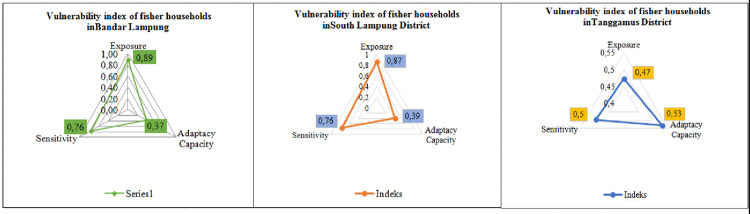
Vulnerability index of fisher households in Bandar Lampung, South Lampung District, and Tanggamus District.

The results of the study [46] revealed that the size of the ship is one factor that impacts the vulnerability of fishermen to climate variability, adopting from the study the ownership and type of ship included in the sensitivity calculation. Fig 3 shows the results of the calculation of the sensitivity level measured from fulfillment food needs, which is 0.60, while boat ownership and boat type are 0.81. Overall, the sensitivity index obtained is quite high at 0.71. Tanggamus Regency is the Regency with the lowest sensitivity of the other regencies, which is 0.49. This suggests that Tanggamus is less sensitive to changes in climate variability than the other two districts. The results of the study show that most traditional fisher households in the three districts utilize part of their catches for consumption and utilize the sale of catches to meet basic daily needs. (start here last night) It is suspected that the level of commercialization of fishing households affects the sensitivity in responding to the impacts of climate variability, but this requires further research. Boat ownership affects fisher households’ sensitivity due to climate change pressures. All respondent fishermen use privately owned boats, which are small and not overnight. Fishermen who use small boats are more sensitive to the phenomenon of climate variability. The results of this study are in line with research [59], where the sensitivity index of fishermen is high at 0.47 because some fishermen depend on meeting their needs from fish catches. The vulnerability index of fisher households to climate variability in the research location is shown in Fig 4.

Studying the livelihood vulnerability of different types of fishers is an important basis for helping fishers rebuild sustainable livelihoods [20]. Based on the results of calculations of the Livelihood Vulnerability Index–Intergovernmental Panel of Climate Change (LVI-IPCC), namely with the formula (exposure—adaptive capacity) x sensitivity. Researchers have computed the vulnerability indices for fishing households in three distinct regions: Bandar Lampung, South Lampung Regency, and Tanggamus Regency. The calculations reveal that the vulnerability index for fishing households is 0.39 in Bandar Lampung, 0.36 in South Lampung Regency, and -0.029 in Tanggamus Regency. These findings indicate that both Bandar Lampung and South Lampung Regency exhibit income vulnerability levels nearing a positive threshold (+1). Tanggamus Regency fishermen do not experience vulnerability with the calculation of the LVI-IPCC value of negative -0.029. The results of this study are similar to research [49], where traditional fishermen are more vulnerable than modern fishermen in Tanggamus Regency. In line with research [47], small fishing households in Dendun Village, Riau Islands, experienced a high vulnerability of 0.29 due to increased extreme weather. The results of research by [60] stated that the coastal fish farming community in Indramayu experienced vulnerability with a high enough value with a vulnerability index of 1.76. It can occur due to tides, land subsidence, and sea level rise. This high vulnerability is caused by increased exposure to shoreline erosion, high-risk perception, limited income, a weak housing structure, and a lack of financial capital [61]. Tanggamus Regency does not experience vulnerability because it can utilize its adaptation strategy by not depending on income sources from fishing activities alone, fishermen in Tanggamus Regency utilize income from outside fishing, namely crop farming, industry, trade and services. This is in line with the results of research by [62] shows that the results of the LVI-IPCC calculation are negative, namely (-0.040), this means that households do not experience vulnerability because farmer households in Kalianyar and Krangkeng Villages, Indramayu Regency, West Java Province can carry out strategies and utilize good adaptation capabilities in dealing with drought phenomena due to climate variability.

The vulnerability experienced by fisher households in Bandar Lampung and South Lampung Regency is due to the fact that almost all fisher households still depend on income sources derived from fishing activities to fulfill their daily needs. Climate variability that occurs, such as erratic rainfall and high waves accompanied by strong winds, results in changes in seasonal patterns, and erratic weather conditions can prevent fishermen from carrying out fishing activities. Traditional capture fishermen in Bandar Lampung, South Lampung Regency, and Tanggamus Regency are classified as small fishermen whose technical skills and managerial capabilities still need to be improved, making it difficult to adjust to climate variability. Climate variability impacts the vulnerability of fisher households because fisher households depend highly on capture fisheries products [63]. It means that an increase in climate variability leads to an increased risk of failure for fishers in fishing activities at sea. If there is a failure in fishing, it impacts the decline in the income of fisher households. It can threaten the success and sustainability of fishermen’s livelihoods. It means that fishermen become vulnerable to changes in climate variability. Research results [64] emphasize that the specificity of a location or region is very important to understand to be assessed appropriately so that vulnerability mitigation can be carried out effectively to improve the welfare of fishermen’s livelihoods in the future. Thus, to ensure the sustainability of fishermen’s livelihoods, it is necessary to know about the condition of the local ecosystem and the surrounding environment.

### Adaptation mechanisms of fishermen households

The adaptation mechanism of individuals or households is built to adjust to their ever-changing surroundings with one goal of survival [65]. Small farmers, who face challenges such as low income and susceptibility to disasters like floods, should actively engage in climate change adaptation, as supported by research findings indicating the rationality of income diversification for their survival. In addition, research results [66] state that adaptations made by farmers and fishermen can significantly increase the happiness and life satisfaction of fishermen. Thus, understanding social vulnerability and community strategies to adapt to environmental change is critical to developing actions to improve conservation and community survival [19]. Table 2 describes the efforts made by traditional capture fisher households in Bandar Lampung, South Lampung District, and Tanggamus District by utilizing their income capital to deal with perceived ecological and economic impacts because of climate variability. Adaptation mechanisms of fisher f households in Bandar Lampung, South Lampung District, and Tanggamus District is shown in Table 3.

**Table 3:** Adaptation mechanism of fisher households in Bandar Lampung, South Lampung District, and Tanggamus District.

Threats	Income Capital	Adaptation Strategy
Ecological Impacts	Physical Capital Natural Capital	• Fishing or coastal fishing • Expanding the catchment area
Economic Impacts	Human Capital Financial Capital Social Capital	• Doing side jobs • Utilize the income of other working family members • Utilize the savings you have • Make loans with third parties • Receive assistance from the government

The adaptation mechanism carried out by fisher households in dealing with ecological impacts is fishing on the coast. During the lean season, when conditions do not allow fishing, some fishermen in Bandar Lampung, South Lampung Regency, and Tanggamus Regency chose to fish or look for fish on the coast. This is done because most respondent fisher households depend on the catch for food. Another strategy is to expand the fishing grounds. Although during the west season the water conditions are bad, some fishermen still carry out fishing activities to the other areas. Most of them will look for new areas that allow them to go to sea and conduct fishing activities. When off fishing, fishermen experience problems in fishing activities as a result of which socio-economic problems arise in their households, in dealing with these problems, households need to develop adequate adaptation strategies called coping strategies [67, 68]. Family coping strategies are behavioral and mental responses to stress by using existing resources in individuals or the surrounding environment [69]. Hence, implementing this coping strategy aims to reduce and manage the emergence of conflicts, internal and external, to improve the lives of fishermen.

The climate variability that occurs impacts the decline in fish catches, which has implications for the decline in the income of fishing households. It has prompted family members of fishers’ households to seek additional employment opportunities to supplement their family income. In addition, some fishermen work on the side as cooperative employees and Quranic teachers. Based on the research results, it is known that fishermen’s wives and children also work as laborers, employees, Quran teachers, household assistants, tailors, Integrated Healthcare Center (Posyandu) cadres, and traders to help increase family income. Other efforts made are by utilizing financial capital and social capital owned to adapt to climate variability. Financial capital is utilizing savings or deposits owned by fisher households. The interview results indicate that specific fishing households actively save by earmarking the proceeds from fish sales, a practice particularly pronounced during the eastern or peak season when they accrue substantial income. The savings can be used as a backup to fulfill basic needs when not at sea. In relation to the coping strategies described earlier, fishermen in Bandar Lampung, South Lampung Regency, and Tanggamus Regency have implemented coping strategies as a response to the problems they face. In line with [67], Resource utilization as a coping strategy for fishing households is carried out by diversifying sources of income through fishing labor activities, aquaculture, and trade and service businesses. This statement is reinforced by the results of research [70], which obtained the same results, namely that households that diversify their income in rural areas have better resilience to climate change.

In terms of social capital, the strategy taken is to utilize loans from third parties and assistance provided by the government. The decline in catches, especially during the lean season, also causes limited capital to carry out fishing operations. This has led some respondent fishermen to borrow from the collector traders with the local term "langan." This is in line with research [47] where small-scale fishermen in Dendun Village, Bintan Regency, borrowed money from collectors to increase the shortage of boat purchase costs and purchase other fishing gear to reduce the decline in income due to climate variability. The loans provided are used to meet the operational costs of going to sea. Fishermen who borrow money from collecting traders (langan) usually have to sell their catches only to the collecting traders. In this case, the collector traders (Langan) also determine the selling price of the fishermen’s catch, usually the selling price to the langan is slightly lower than the selling price in the market. Borrowing is also done based on business experience; usually, the longer the business experience, the more access to loans, this is in line with [71] and [72] that business experience and access to loans have a positive significant relationship. The assistance received by fishermen is in the form of cash and net fishing equipment. Other adaptation strategies, namely reducing risks associated with fishing activities, strengthening social cohesion, increasing fishermen’s knowledge about climate change, increasing technical skills and management capabilities of alternative economic businesses, increasing fishermen’s participation in climate change adaptation planning, and increasing fishermen’s access to resources. credit [7].

## Conclusion

The main income of traditional fishermen’s households in Bandar Lampung, South Lampung Regency and Tanggamus Regency comes from sources related to the fishing industry. This shows how dependent the source of income of fishermen’s households is on fish catches. The climate variability that occurs creates vulnerability in the livelihoods of fishing households. The level of vulnerability is determined by the adaptability, sensitivity and exposure of each type of fisherman, requiring appropriate policies to support the sustainability of their livelihoods.

The income structure of traditional capture fisher households in Bandar Lampung, South Lampung Regency, and Tanggamus Regency is still dominated by income sources derived from fishing business activities. Tanggamus Regency contributes the largest source of income from capture fisheries, while South Lampung Regency contributes the smallest income. This shows that capture fisheries business activities are very important as a source of livelihood for fishing communities in Lampung Province so that their existence and sustainability need to be maintained.

Climate variability has disrupted the livelihood system of fisher households, making them vulnerable to fluctuations in their income sources. The calculation results show that traditional capture fisher households in Bandar Lampung City and South Lampung Regency experience income vulnerability with a positive LVI-IPCC value, while Tanggamus Regency does not experience vulnerability with a negative vulnerability index. The difference in income vulnerability is due to the difference of average boat ownership and boat capacity. An important implication of this finding is the importance of fishing gear assistance by the government for fishermen should pay attention to the scale of boat ownership and the type of boat with higher capacity.

Adaptation mechanisms carried out by fishermen in Bandar Lampung, South Lampung Regency, and Tanggamus Regency as a response to climate variability, including by fishing on the coast, expanding fish catch areas, doing side jobs and utilizing family members’ income, utilizing savings, making loans of collectors (langan), and utilizing assistance from the government.

Adaptation strategies to climate variability are very important because they affect the level and stability of fishermen’s income. In addition, the income diversification that fishermen have done is a good step to increase and maintain the stability of their household income. The government, in addition to providing boat and fishing gear assistance, needs to encourage fish farming activities on the coast, develop off-fishing and non-fishing activities in coastal villages, and build fisheries infrastructure in coastal areas that support business activities both on fishing, off fishing and non-fishing.

Direct assistance in the form of cash transfers as well as boat and fishing gear assistance according to the needs of fishermen is urgent because they really need it to survive and revive activities as capture fishermen, fish and other commodity cultivation, and non-capture fishery activities. However, some programs that are considered strategic are increasing the capacity of fishermen’s human resources both in terms of technical skills and managerial capabilities, strengthening fishermen’s institutions at the locality level in mitigating and adapting to climate variability, and empowerment of fishing communities and their families in the development of productive economic enterprises to increase alternative sources of income.
